# ﻿Ardisiacrenatasubsp.mukdahanensis, a new subspecies of Primulaceae from Thailand

**DOI:** 10.3897/phytokeys.247.126743

**Published:** 2024-09-26

**Authors:** Wilawan Promprom, Phukphon Munglue, Wannachai Chatan

**Affiliations:** 1 Department of Biology, Faculty of Science, Mahasarakham University, Kantharawichai District, Mahasarakham Province, 44150, Thailand; 2 Plant and Innovation Research Unit, Mahasarakham University, Mahasarakham, 44150, Thailand; 3 Program of Biology, Faculty of Science, Ubon Ratchathani Rajabhat University, Ubon Ratchathani 34000, Thailand

**Keywords:** Classification, diversity, Myrsinaceae, north-eastern Thailand, pollen, taxonomy

## Abstract

A new subspecies, ArdisiacrenataSimssubsp.mukdahanensis Chatan & Promprom (Primulaceae), is described from Mukdahan Province, Thailand. This discovery was made during field studies conducted from 2021 to 2023, during which the authors collected and analysed specimens, comparing them with relevant taxonomic literature and herbarium collections. A.crenatasubsp.mukdahanensis is distinct from its closest relative, A.crenatasubsp.crassinervosa by a combination of morphological traits, such as moderately dense minute hairs on young shoots, abaxial side of young lamina and calyx, peduncles and pedicels; larger flowers (7.0–7.5 mm) that are pure white or pinkish; larger fruits (7–8 mm) and absence of glandular punctation in organs such as lamina, calyx, corolla, anther and fruit. This new subspecies grows in slightly dense dry evergreen forests or open areas near streams and is preliminarily assigned to the Data Deficient (DD) category according to IUCN guidelines.

## ﻿Introduction

*Ardisia* Swartz is a genus within the family Primulaceae, comprising approximately 500 to 735 species worldwide, with the majority found in Asia (Mao and Hu 2013; POWO 2024). Traditionally, *Ardisia* was classified within the Myrsinaceae by several taxonomists, including Larsen and Hu (1996, 2001) and Hu and Vidal (2004). In Thailand, the taxonomic study reported 72 species of *Ardisia* in the Flora of Thailand (Larsen and Hu 1996). Recent updates to the Flora of Thailand have included the description of a new subspecies of *A.crenata* Sims (Chatan and Promprom 2017b), the identification of new species, lectotypification and other taxonomic changes (Larsen and Hu 2001; Chatan and Promprom 2017a; Suparman et al. 2021).

During the flora exploration in Mukdahan Province, between 2021 and 2023, the authors discovered an undescribed taxon and brought it back to the laboratory at Mahasarakham University for further analysis. After carefully examining and comparing our findings with relevant taxonomic literature and herbarium collections from major herbaria in Thailand and Europe, we concluded that the undescribed taxon belongs to subgenus Crispardisia. This conclusion is based on the presence of nodules on sinuses on the crenate leaves. Additionally, the undescribed taxon is closely related to *A.crenata*, based on their crenate margins, marginal glands, flowering branches close to the apex of the stem or branch, mostly simple inflorescences and short calyx. Amongst the subspecies of *A.crenata*, it mostly resembles A.crenatasubsp.crassinervosa (E. Walker) C.M.Hu & J.E.Vidal, as both share similar morphological characteristics, such as subcoriaceous and distinct-veined leaf blades, mostly sub-umbellate inflorescences and predominantly broadly ovate calyx lobes. These similarities suggest that the two subspecies are closely related, differing mainly in more specific morphological features. The following description is based on observations of both dried and fresh specimens, accompanied by illustrations of this newly-recognised taxon.

In the subgenus Crispardisia, pollen morphological characteristics could provide information for dividing sections and intra-sectional groups within *Ardisia* (Zhang et al. 2007). In this study, pollen morphology is also examined to provide additional insights into this subgenus.

## ﻿Materials and methods

Field trips were conducted in Mukdahan Province between 2021 and 2023. Some mature plants of the undescribed taxon from these fields were planted in the third author’s home garden in Kantharawichai District, Maha Sarakham Province, Thailand, for further morphological observation. We collected specimens including the type and studied the morphological characters of the living collection. We meticulously examined and compared our findings with relevant taxonomic literature including works by Sims (1817), Walker (1940), Chen and Pipoly (1996), Hu and Vidal (1996), Larsen and Hu (1996), Hu (1999), Larsen and Hu (2001), Hu and Vidal (2004), Zhang et al. (2007) and Chatan and Promprom (2017b).

The specimens were compared with herbarium collections from major herbaria in Thailand, including the Forest Herbarium, Department of National Parks, Wildlife and Plant Conservation (**BKF**) and the Bangkok Herbarium (BK). Additionally, images of other herbarium specimens of related species were examined from the databases of international collections, such as the Kew Herbarium (http://apps.kew.org/herbcat/gotoHomePage.do), the Muséum national d’Histoire naturelle (P) (https://science.mnhn.fr/all/search) and the JSTOR Global Plants database (http://plants.jstor.org/).

About 20 pollen grains of A.crenatasubsp.mukdahanensis were examined by using Erdtman’s method (Erdtman 1972). The pollen grains were dehydrated through a series of ethanol solutions at concentrations of 70%, 80%, 95% and 100%, with each step taking 5 minutes. After dehydration, the pollen was left to air-dry overnight at room temperature. The dried pollen was then mounted on to aluminium panels, which were attached to stubs using carbon tape. Then, the pollen grains were analysed using a scanning electron microscope (SEM) (Hitachi, TM-4000plus, Hitachi High-Tech, Tokyo, Japan) at the Laboratory Equipment Center in Mahasarakham University’s Division of Research Facilitation and Dissemination. The analysis was conducted following Erdtman’s pollen shape classification and terminology (Erdtman 1972).

## ﻿Taxonomic treatment

### ﻿ Ardisia crenata Sims subsp. mukdahanensis

Taxon classificationPlantaeEricalesPrimulaceae

Chatan & Promprom sp. nov.

0E978B55-6A83-593B-A640-A70D0F30C5B2

urn:lsid:ipni.org:names:77349016-1

Figs 1 , 2 , 3 , 4

#### Type.

Thailand • Dong Luang District, Mukdahan Province, alt. 220–250 m, 16°46'23"N, 104°21'58"E, 10 November 2023 (fl.), *W. Chatan 2886* (holotype: BKF!; isotype: BK!).

#### Diagnosis.

Ardisiacrenatasubsp.mukdahanensis is closely related to subsp. crassinervosa. However, the morphological distinctions of the new subspecies from the latter are as follows: moderately dense, minute hairs are present on the surfaces of young shoots, the abaxial side of the lamina (with very few or sparse hairs on older ones), peduncles, pedicels and the abaxial side of the calyx of the new subspecies, while they are absent in the latter. It has larger flowers (7.0–7.5 mm) that are typically pure white or pinkish. The fruits are mostly larger (7–8 mm in diameter) and glandular punctation is absent in the lamina, calyx, corolla, anther and fruit in the new subspecies, whereas they are present in the latter.

**Figure 1.**
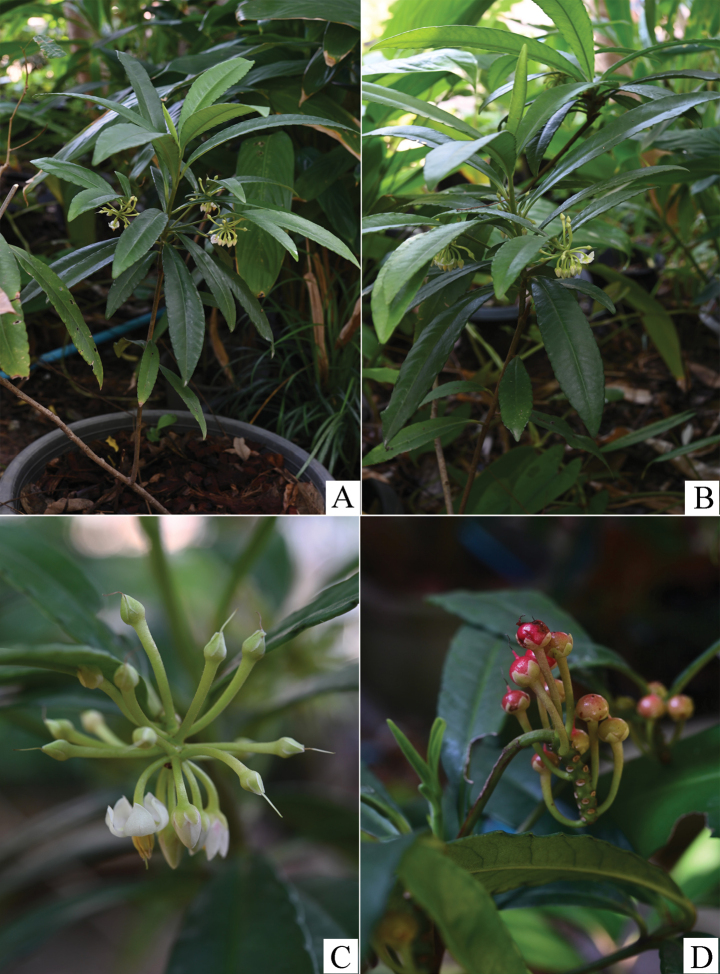
Ardisiacrenatasubsp.mukdahanensis**A, B** habit **C** inflorescence **D** infructescence (Image by Wannachai Chatan).

#### Description.

Shrubs 40–100 cm high; branchlets slender, terete or angular, striate, generally each branch bearing more than 10 leaves. ***Leaves*** alternate, petioles 0.4–2.0 cm long; lamina subcoriaceous, oblanceolate or oblanceolate-oblong, 5–20 × 1.5–4 cm; base cuneate or attenuate; margin distinctly crenate and recurved, with large marginal glands on sinuses; adaxial surface glabrous or glabrescent, glandular dots absent; apices mostly obtuse, rarely acute; abaxial surface with very few minute hairs and sparsely hairs on the mid-rib, glandular dots absent; veins distinct; intramarginal veins present at 1.5–2.5 mm from the lamina edge at the middle between the marginal glands and those veins close to the margin by joining to the marginal glands. ***Inflorescences*** sub-umbellate or corymbiform, mostly simple or occasionally compound, terminal on branchlets; peduncles 0.4–0.5 cm long moderately dense minute hairs on the surface; bract oblong and V-shaped, 8–11 × 2.0–2.5 mm, primary rachis 2–6 mm long; pedicels 8–10 mm long, cylindrical, green, surface with moderate hairs. ***Calyx*** of 5-lobes, split almost to the base, pale green on both surfaces, distinctly imbricate at base; lobes broadly ovate, 3.0–3.5 × 2.5–3.0 mm, glandular-dots absent, apices acute or obtuse, margin entire and translucent, adaxially glabrous, abaxially covered with moderately dense minute hairs. ***Corolla*** of 5-lobes, connate at about 1 mm at the base, pure white or pinkish, sometimes pinkish only at base and centre, thick and succulent, lobes convolute in bud, broadly ovate, concavo-convex, 7.0–7.5 × 4.5–5 mm, glandular-dots absent on both surfaces, apex mucronate with curved mucro. ***Stamen*** 5; filament whitish, ca. 1 mm long; anther lanceolate, yellow, 5.0–5.5 × 1.8–2.1 mm, apex acute, glandular dots absent. ***Gynoecium*** length is longer than the stamen; ovary globose, 1.4–1.6 mm diameter, green, glabrous, 7 locules, 1 ovule in each locule, ovules in 1-series; styles about 4.5–5.0 mm long, irregularly curved and narrow to the apex, sparsely minute hairs on the lower half; stigma minute. ***Fruits*** young green, mature red, globose, 7–8 mm diameter, glandular dots absent. ***Seed*** globular, 4.0–4.5 mm diameter, brown.

**Figure 2.**
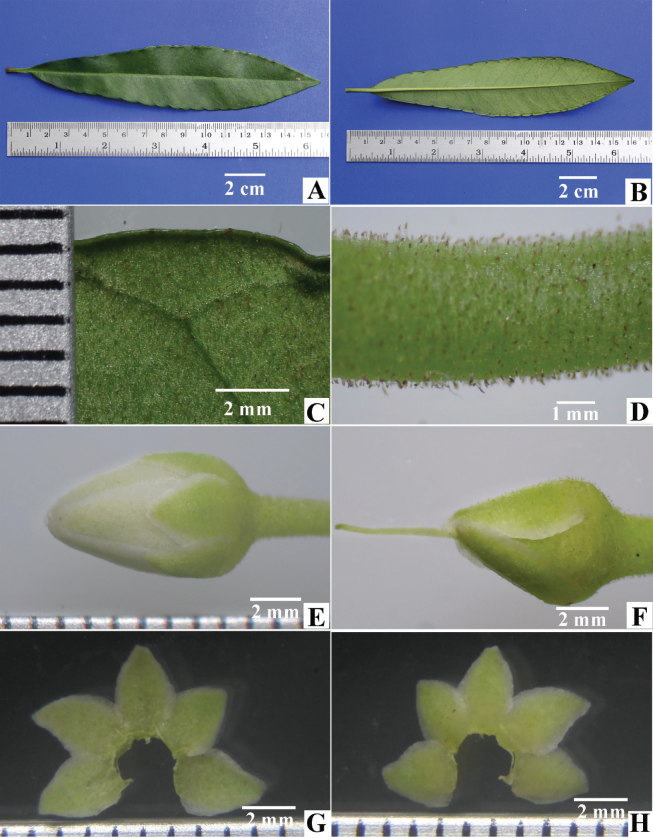
Ardisiacrenatasubsp.mukdahanensis**A** leaf, adaxial side **B** leaf, abaxial side **C** intramarginal vein showing the distance from the margin **D** peduncle **E** flower bud **F** flower bud with corolla removed **G** dissected calyx, adaxial side **H** dissected calyx, abaxial side.

#### Additional specimen examined.

Thailand • Dong Luang District, Mukdahan Province: Phu Pha Yol National Park, alt. 220–250 m, 16°46'42.3"N, 104°21'25.6"E, 1 September 2021 (fr.), *W. Chatan 2504* (paratype: BKF).

**Figure 3.**
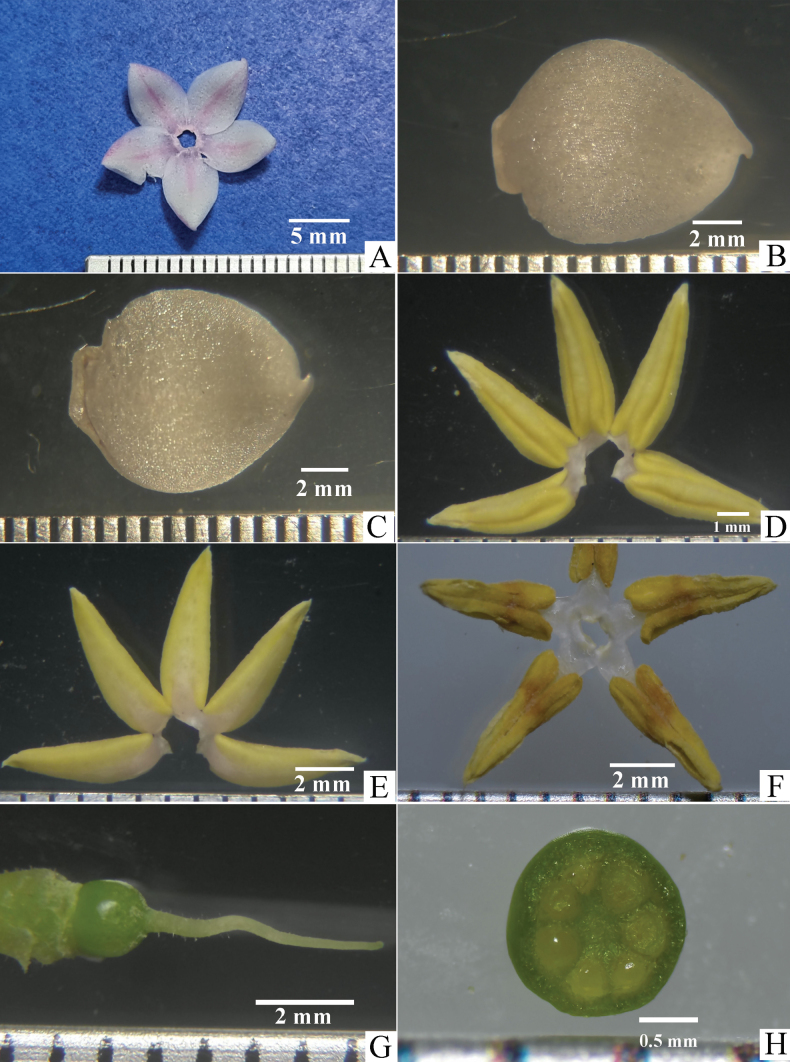
Ardisiacrenatasubsp.mukdahanensis**A** dissected corolla **B** corolla-lobe, adaxial side **C** corolla-lobe, abaxial side **D** androecium, adaxial side **E** androecium, abaxial **F** androecium with opened anthers, adaxial **G** gynoecium **H** dissected ovary showing the ovules (X.S.).

**Figure 4.**
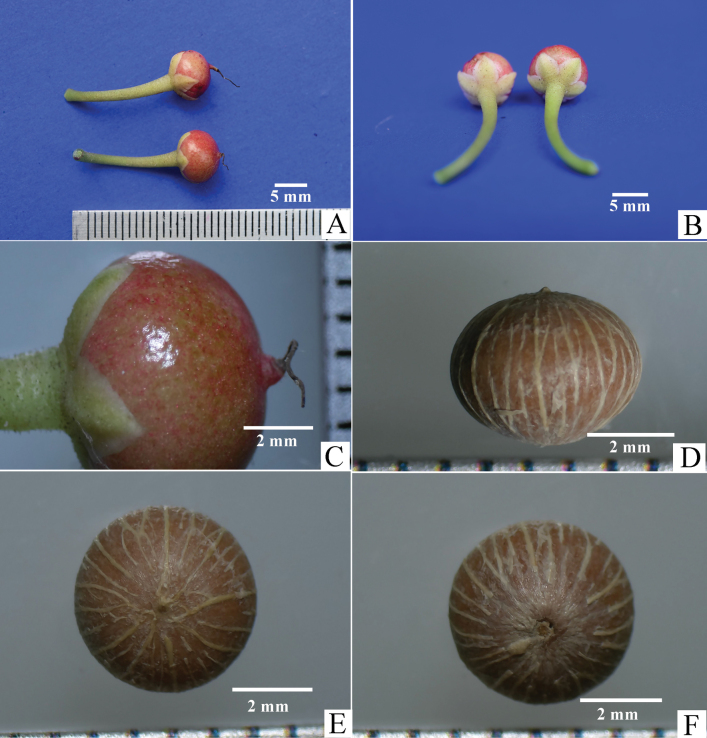
Ardisiacrenatasubsp.mukdahanensis**A** fruits, lateral view **B** fruits, bottom view **C** fruit’s surface showing its colour and lacks glandular punctation **D–F** seed, lateral, aerial and bottom views, respectively.

#### Distribution.

**Figure 5.**
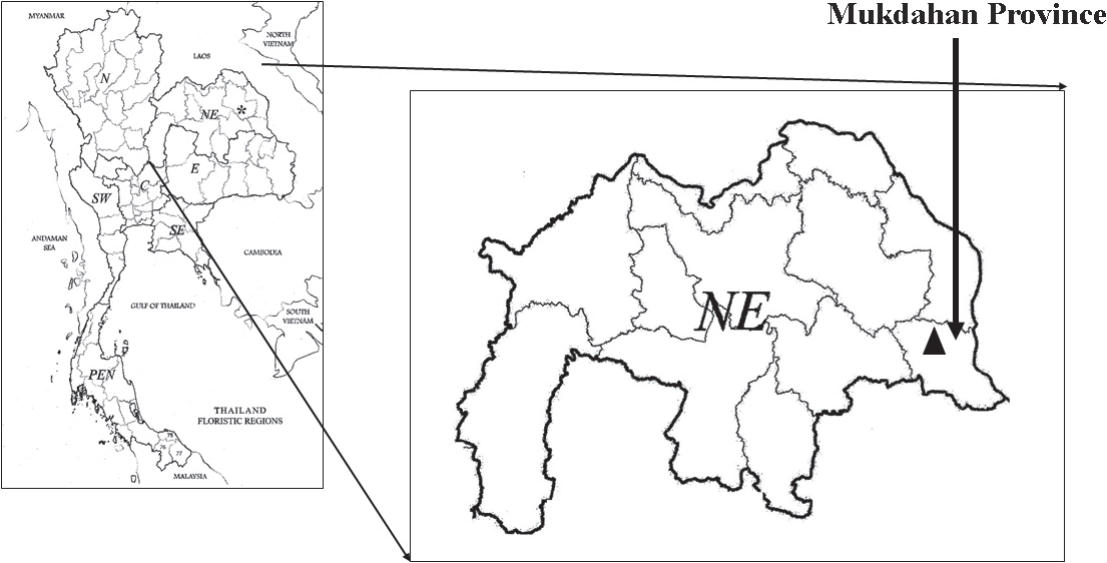
Distribution of Ardisiacrenatasubsp.mukdahanensis at Dong Luang District, Mukdahan Province, Thailand.

Ardisiacrenatasubsp.mukdahanensis is an endemic to Thailand. So far, it has been only found in the type locality in Dong Luang District, Mukdahan Province. Its distribution is shown in Fig. 5.

#### Ecology.

It mostly grows in slightly dense dry evergreen forests or open areas and usually grows near the stream. Sometimes it grows in dry-dipterocarp forests.

#### Phenology.

Flowering in May to November and fruiting in June to February.

#### Vernacular name.

Takai Kao.

#### Etymology.

The specific epithet ‘*mukdahanensis*’ refers to its type locality, the Mukdahan Province, in the northeast of Thailand.

#### Provisional conservation status.

Currently, A.crenatasubsp.mukdahanensis is known only from its type locality. Comprehensive fieldwork is needed to conduct a thorough conservation assessment. Therefore, the species is classified as Data Deficient (DD) according to the Guidelines for using the IUCN Red List Categories and Criteria (IUCN Standards and Petitions Committee 2024).

#### Palynology.

**Figure 6.**
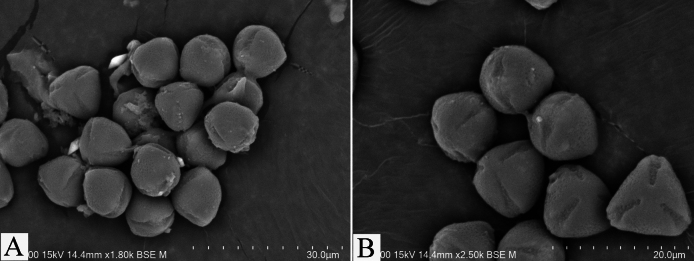
Scanning electron microscopy (SEM) photomicrographs of pollen morphology of Ardisiacrenatasubsp.mukdahanensis**A**, **B** show the pollen’s morphology at different magnifications.

The pollen grains of A.crenatasubsp.mukdahanensis are monads, semi-angular in polar shape, oblate-spheroidal in equatorial shape, small size, 11.50 ± 1.30 µm in equatorial axis, 10.20 ± 1.20 µm in polar, radially symmetrical, isopolar, tricolpate, separate apertures at the pollen pole, exine sculpturing foveolate-reticulate, perforate (Fig. 6).

#### Notes.

In the most recent update of classification, *A.crenata* was classified into three subspecies: A.crenatasubsp.crenata, A.crenatasubsp.crassinervosa and A.crenatasubsp.obtusifolia (Chatan and Promprom 2017b). The discovery of A.crenatasubsp.mukdahanensis has expanded this to four subspecies within *A.crenata*. Ardisiacrenatasubsp.mukdahanensis is most closely related to A.crenatasubsp.crassinervosa, but it differs by having the following distinct characteristics: moderately dense, minute hairs present on the surfaces of young shoots, the abaxial side of the lamina (with very few or sparse hairs on older ones), peduncles, pedicels and the abaxial side of the calyx, whereas these hairs are absent in the latter. Additionally, A.crenatasubsp.mukdahanensis typically has larger flowers (7.0–7.5 mm vs. 4–5 mm) that are pure white or pinkish (vs. pink or purplish), larger fruits (7–8 mm vs. ca. 5 mm in diameter) and lack glandular punctation in the lamina, calyx, corolla, anther and fruit, which are present in A.crenatasubsp.crassinervosa (Figs 1–4). Details of the morphological differences are shown in Table 1.

**Table 1.** Distinguishing features between Ardisiacrenatasubsp.mukdahanensis and A.crenatasubsp.crassinervosa.

Feature	A.crenatasubsp.mukdahanensis	A.crenatasubsp.crassinervosa
Plant height	40–100 cm tall	100 cm tall
Petiole length	0.4–2.0 cm long	up to 3 mm long
Indumentum	moderately dense, minute hairs present on the surfaces of young shoots, abaxial side of lamina, peduncles, pedicels and abaxial side of calyx-lobes	moderately dense, minute hairs lacking on the surfaces of young shoots, abaxial side of lamina, peduncles, pedicels and the abaxial side of the calyx-lobes
Lamina	5–20 × 1.5–4.0 cm, subcoriaceous	4–10 × 1.5–4.0 cm, subcoriaceous or coriaceous
Leaf apex	mostly obtuse, rarely acute	acute
Lamina surface	without glandular punctations on both surfaces	with or without black punctations beneath
Inflorescences	subumbellate or corymbiform, mostly simple or occasionally compound	subumbellate, simple
Pedicels	8–10 mm long, cylindrical	ca. 10 mm long, somewhat flattened
Flowers	pure white or pinkish, 7.0–7.5 mm long	pink to purplish, 4–5 mm long
Calyx lobes	broadly ovate, 3.0–3.5 mm long, glandular dots absent	broadly ovate or suborbicular, 2.5–3 mm long, glandular dots present or obscure
Corolla lobes	glandular dots absent on both surfaces	glandular dots present abaxially
Anthers	lanceolate, glandular dots absent	lanceolate, glandular dots present
Fruits	7–8 mm diameter, without glandular dots	ca. 5 mm in diameter, with prominent glandular dots

Several key characteristics can distinguish the new subspecies from the other subspecies. Compared to A.crenatasubsp.crenata, the new subspecies has a subcoriaceous lamina (vs. chartaceous or subcoriaceous), a mostly obtuse lamina apex (vs. mostly acute or acuminate) and broadly ovate calyx-lobes (vs. ovate or ovate-oblong). In contrast to A.crenatasubsp.obtusifolia, the new subspecies differs in having a subcoriaceous lamina (vs. highly coriaceous), an oblanceolate or oblanceolate-oblong leaf shape (vs. spathulate, narrowly elliptic or oblanceolate) and distinctly crenate and recurved on leaf margin (vs. sub-entire or shallowly crenate, undulate). In summary, the new subspecies is distinguished from A.crenatasubsp.crenata by its lamina texture, shape of apex and sepal and from A.crenatasubsp.obtusifolia by its lamina texture, shape and margin (Figs 1, 2).

Zhang et al. (2007) examined the pollen grains of 23 species of Ardisiasubgen.Crispardisia from China. The pollen grains of the studied taxa are subspheroidal to suboblate in shape and 3-colporate, forming syncolpate (except for *A.faberi*). Four pollen grain types were identified: type I (with foveolate-reticulate sculpture), type II (with finely reticulate sculpture), type III (with rugulate sculpture) and type IV (with finely granulate sculpture with spines). The pollen grains of *A.crenata* (based on the two samples of *A.crenata*, not identified at the infraspecific level) and one sample of *A.crassinervosa* E.Walker (subsp.crassinervosa) are classified as types I. Similar to these two studied taxa, the new subspecies has type I of pollen grain sculpturing. Additionally, the new subspecies are distinct amongst the most studied *Ardisia* taxa in terms of having tricolpate pollen with separate apertures at the pollen pole. Based on this pollen morphology, our collection is best recognised as a new subspecies of *A.crenata*, though studies on pollen morphology and other taxonomic characteristics are needed for further clarification of its taxonomic status.

## Supplementary Material

XML Treatment for Ardisia crenata Sims subsp. mukdahanensis

## ﻿Additional information

### Author ORCIDs

Wilawan Promprom https://orcid.org/0000-0001-8821-2267

Phukphon Munglue https://orcid.org/0000-0001-7247-2873

Wannachai Chatan https://orcid.org/0000-0002-3941-7626

### Data availability

All of the data that support the findings of this study are available in the main text.
